# Nitrogen assimilation by *E. coli* in the mammalian intestine

**DOI:** 10.1128/mbio.00025-24

**Published:** 2024-02-21

**Authors:** Sudhir Doranga, Tyrrell Conway

**Affiliations:** 1 Department of Microbiology and Molecular Genetics, Oklahoma State University, Stillwater, Oklahoma, USA; 2 Department of Cardiovascular and Metabolic Sciences, Cleveland Clinic, Cleveland, Ohio, USA; University of Hawaii at Manoa, Honolulu, Hawaii, USA

**Keywords:** *E. coli*, intestinal colonization, nitrogen assimilation, gut microbiome

## Abstract

Nitrogen is an essential element for all living organisms, including *Escherichia coli*. Potential nitrogen sources are abundant in the intestine, but knowledge of those used specifically by *E. coli* to colonize remains limited. Here, we sought to determine the specific nitrogen sources used by *E. coli* to colonize the streptomycin-treated mouse intestine. We began by investigating whether nitrogen is limiting in the intestine. The NtrBC two-component system upregulates approximately 100 genes in response to nitrogen limitation. We showed that NtrBC is crucial for *E. coli* colonization, although most genes of the NtrBC regulon are not induced, which indicates that nitrogen is not limiting in the intestine. RNA-seq identified upregulated genes in colonized *E. coli* involved in transport and catabolism of seven amino acids, dipeptides and tripeptides, purines, pyrimidines, urea, and ethanolamine. Competitive colonization experiments revealed that L-serine, N-acetylneuraminic acid, N-acetylglucosamine, and di- and tripeptides serve as nitrogen sources for *E. coli* in the intestine. Furthermore, the colonization defect of a L-serine deaminase mutant was rescued by excess nitrogen in the drinking water but not by an excess of carbon and energy, demonstrating that L-serine serves primarily as a nitrogen source. Similar rescue experiments showed that N-acetylneuraminic acid serves as both a carbon and nitrogen source. To a minor extent, aspartate and ammonia also serve as nitrogen sources. Overall, these findings demonstrate that *E. coli* utilizes multiple nitrogen sources for successful colonization of the mouse intestine, the most important of which is L-serine.

**IMPORTANCE:**

While much is known about the carbon and energy sources that are used by *E. coli* to colonize the mammalian intestine, very little is known about the sources of nitrogen. Interrogation of colonized *E. coli* by RNA-seq revealed that nitrogen is not limiting, indicating an abundance of nitrogen sources in the intestine. Pathways for assimilation of nitrogen from several amino acids, dipeptides and tripeptides, purines, pyrimidines, urea, and ethanolamine were induced in mice. Competitive colonization assays confirmed that mutants lacking catabolic pathways for L-serine, N-acetylneuraminic acid, N-acetylglucosamine, and di- and tripeptides had colonization defects. Rescue experiments in mice showed that L-serine serves primarily as a nitrogen source, whereas N-acetylneuraminic acid provides both carbon and nitrogen. Of the many nitrogen assimilation mutants tested, the largest colonization defect was for an L-serine deaminase mutant, which demonstrates L-serine is the most important nitrogen source for colonized *E. coli*.

## INTRODUCTION

*Escherichia coli*, possibly the best understood of all model organisms, is a ubiquitous colonizer of the mammalian intestine (1). However, the precise mechanisms of colonization are not fully understood. To stably colonize*, E. coli* must successfully compete with the resident microbiota for nutrients that permit it to grow faster than the turnover rate of luminal contents (1, 2). Studies investigating *E. coli* metabolism in the intestinal mucus layer revealed that *E. coli* K-12 strain relies on arabinose, fucose, gluconate, N-acetylglucosamine (NAG), and N-acetylneuraminic acid (NANA) as carbon and energy sources (3, 4).

In addition to carbon, nitrogen is an essential element for all living organisms as a constituent of amino acids and nucleic acids (5). Nitrogen is also present in many intermediary metabolites (6). In bacteria, nitrogen also is a constituent of peptidoglycan precursors (7). However, the mechanisms by which *E. coli* assimilates nitrogen in the gut are not known. Ammonia is the preferred nitrogen source for *E. coli in vitro*, as it supports the fastest growth rate (8, 9). Substituting ammonia with an amino acid as the sole nitrogen source slows growth (9). Nevertheless, under aerobic conditions, L-aspartate, L-asparagine, and L-glutamine serve as sole nitrogen sources (10). Schubert et al. demonstrated that *E. coli* can also grow anaerobically *in vitro* with aspartate as the sole nitrogen source (11). Furthermore, aspartate is a source of fumarate that supports anaerobic respiration in colonized *E. coli* and *Salmonella enterica* serovar Typhimurium (11, 12). Bertin et al. (13) reported that *E. coli* O157:H7 utilizes aspartate to grow in bovine intestinal contents. Besides aspartate, a few other potential nitrogen sources have been studied. In an induced colitis mouse model, pathogenic *E. coli* and *Citrobacter rodentium* were found to utilize L-serine to maximize their competitive fitness (14). However, it is not known whether these pathogens utilize L-serine as a carbon source, a nitrogen source, or both. *S*. Typhimurium utilizes fructose-asparagine for carbon and nitrogen during growth *in vitro* (15, 16). The ethanolamine utilization pathway (*eut* gene cluster) is upregulated in enterohemorrhagic *E. coli* (EHEC) when grown *in vitro* on bovine intestinal contents (17). Also, ethanolamine can serve as a sole nitrogen source but not as a sole carbon source *in vitro*, suggesting that EHEC can acquire nitrogen from ethanolamine in the intestine (17). It has been reported that uropathogenic *E. coli* can use ethanolamine as a source of both carbon and nitrogen (18). Beyond these limited findings, there is little knowledge of the nitrogen sources used by *E. coli* to colonize.

Diet and endogenous compounds are major sources of nitrogen in the intestine (19–21). Ammonia, amino acids, proteins, peptides, polyamines, purines, pyrimidines, and urea are potential nitrogen sources for *E. coli* in the gut (11, 17, 19, 22–24). While it has been reported that nitrogen availability is limited in the intestine due to host utilization of dietary nutrients (21), it remains unclear whether nitrogen is limiting for colonized *E. coli*. Thus, we aimed to assess whether nitrogen is limiting for colonization and identify specific nutrients that serve as nitrogen sources.

In this study, RNA sequencing demonstrated that genes encoding amino acid biosynthetic pathways were downregulated in colonized *E. coli* compared to *in vitro* cultures in minimal medium with ammonia as the sole nitrogen source, confirming an abundance of amino acids in the intestine. Genes involved in assimilation of amino acids, di- and tripeptides, urea, ethanolamine, etc., were upregulated, suggesting their role in nitrogen supply to *E. coli*. Competitive colonization experiments between *E. coli* MG1655 wild type and knockout mutants in nitrogen assimilation pathways revealed that *E. coli* relies on a few compounds as nitrogen sources, including L-serine, NAG, NANA, and di- and tripeptides. Additional experiments designed to rescue knockout mutants during competitive colonization confirmed that L-serine serves primarily as a nitrogen source, while NANA provides both nitrogen and carbon. While not limiting individually, aspartate and ammonia also contribute to nitrogen assimilation by *E. coli* in the intestine. Our findings suggest a strategy employed by *E. coli* to gain competitive fitness and persistently colonize the intestine by simultaneously utilizing multiple nitrogen sources available in the mucus layer, beginning with L-serine.

## RESULTS

### Nitrogen regulation is important for *E. coli* colonization in the intestine

**Fig 1:**
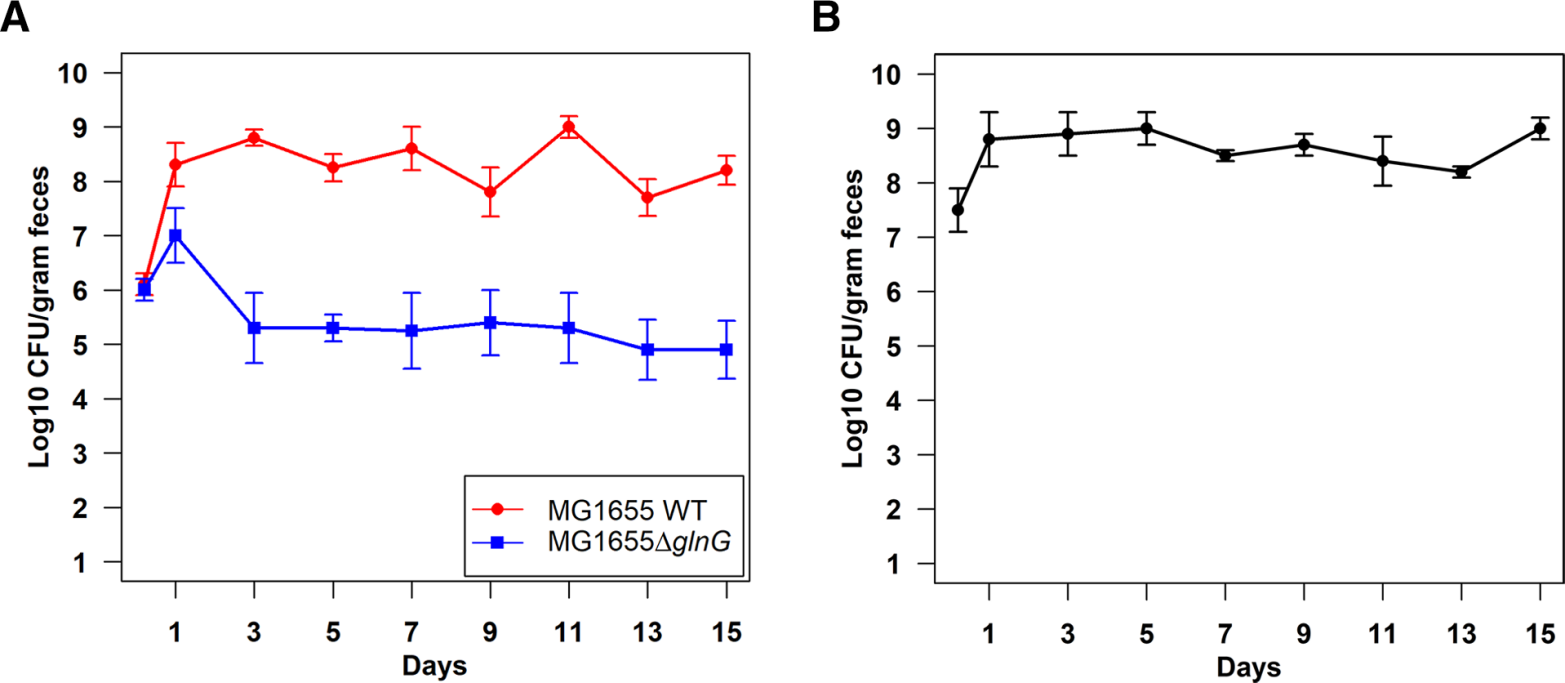
Colonization of *E. coli* MG1655 Str^R^ ∆*glnG::cam* in the mouse intestine.** (A)** Competitive colonization of the mouse intestine by *E. coli* MG1655 Str^R^ Nal^R^ wild type and *E. coli* MG1655 Str^R^ ∆*glnG::cam*. Two sets of three CD-1 male mice were fed 10**^5^** CFU each of the two strains. At the indicated times, fecal samples were collected, homogenized, diluted, and plated. Error bars represent standard errors of the pooled data of log_10_ mean CFU/g of feces. (**B)** Monocolonization of *E. coli* MG1655 Str^R^ ∆*glnG::cam* in the mouse intestine. A set of three CD-1 male mice were fed 10**^5^** CFU of *E. coli* MG1655 Str**^R^** ∆*glnG::cam* and fecal samples were enumerated at indicated time points.

NtrC (product of *glnG*) regulates approximately 100 genes involved in nitrogen assimilation (25). Here, we sought to determine whether regulation by NtrC is important for *E. coli* colonization. *E. coli* MG1655 Str^R^ ∆*glnG::cam* was outcompeted by the wild type by >3 log_10_-fold (Fig. 1A). However, the *glnG* mutant colonized at ~10^9^ CFU/g of feces when fed alone (Fig. 1B). It was previously determined that *glnG* mutants grow poorly compared to the wild type, suggesting that *glnG* is important for fitness even under nitrogen-rich culture conditions (26). This suggests that nitrogen regulation is an important fitness determinant for competitive colonization of the intestine.

### NtrC-dependent genes are downregulated in colonized *E. coli*, indicating nitrogen is not limiting

**Fig 2:**
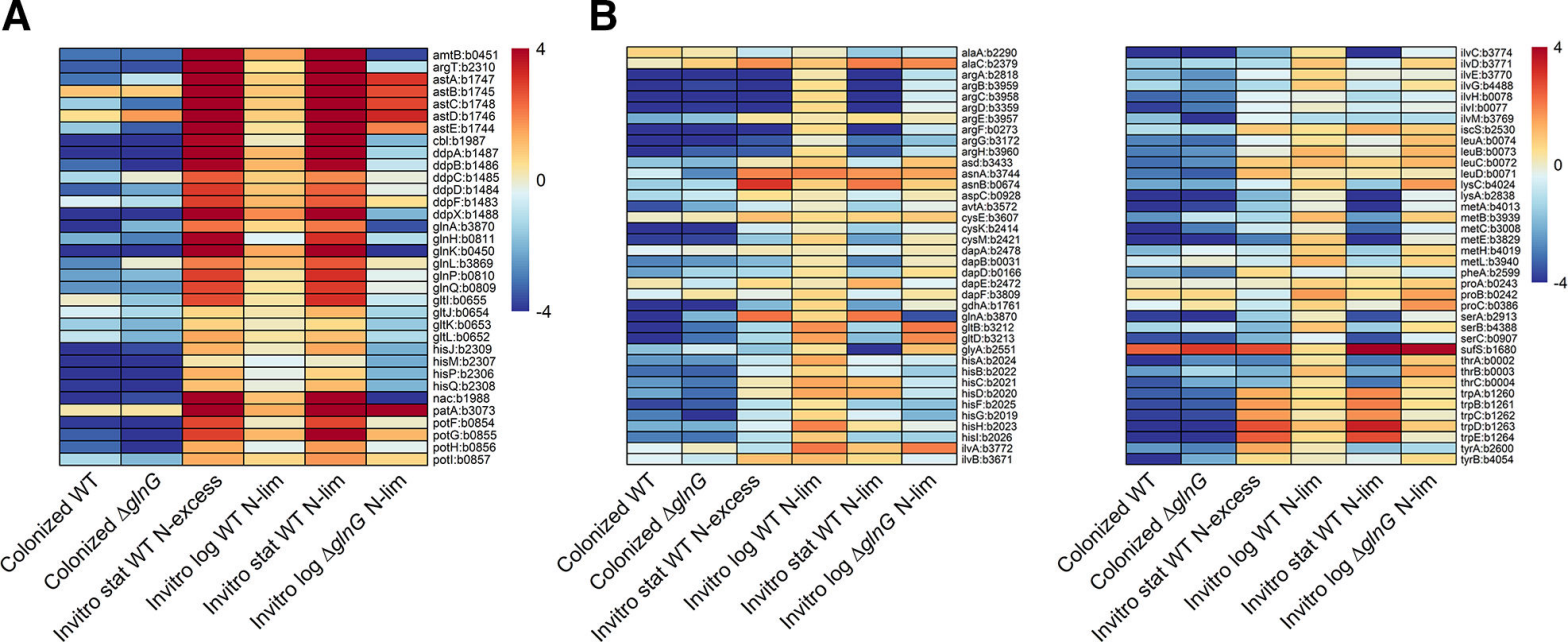
(A) Heatmap showing expression of *E. coli* MG1655 genes controlled by NtrC under different conditions, compared to gene expression when grown in MOPS minimal medium containing 0.2% glucose and 10 mM NH_4_Cl. (**B)** Heatmap demonstrating expression of *E. coli* MG1655 amino acid biosynthesis genes under different conditions compared to expression when grown in MOPS minimal medium containing 0.2% glucose and 10 mM NH_4_Cl. Six different conditions are compared to control conditions: (i) colonized wild type (WT): *E. coli* MG1655 wild type colonized in the mouse cecum compared to log-phase culture; (ii) colonized *∆glnG*: *E. coli* MG1655 *∆glnG* colonized in the mouse cecum compared to log-phase culture; (iii) *in vitro* stat WT N-excess: *in vitro* stationary-phase culture of wild type grown with excess nitrogen (10 mM NH_4_Cl) compared to log-phase culture; (iv) *in vitro* log WT N-lim: *in vitro* log-phase culture of wild-type *E. coli* MG1655 grown with limited nitrogen (3 mM NH_4_Cl) compared to log-phase culture; (v) *in vitro* stat WT N-lim: *in vitro* stationary-phase culture of wild-type *E. coli* MG1655 grown with limited nitrogen (3 mM NH_4_Cl) compared to log-phase culture; (vi) *in vitro* log *∆glnG* N-lim: *in vitro* log-phase culture of ∆*glnG* mutant grown with limited nitrogen (3 mM NH_4_Cl) compared to log-phase culture.

High-throughput liquid chromatography-tandem mass spectrometry revealed an abundance of amino acids in the intestinal contents of mice (11). However, another report indicated that nitrogen is limiting for the microbiota of the large intestines of 30 mammalian species (21). It was not yet known if nitrogen is limiting for *E. coli* in the streptomycin-treated mouse gut. To assess whether NtrC-dependent genes are induced in colonized *E. coli*, we sequenced RNA from cecal mucus of mice colonized separately with *E. coli* MG1655 Str^R^ Nal^R^ wild type or *E. coli* MG1655 Str^R^ ∆*glnG::cam.* We also sequenced RNA from log-phase and stationary-phase cultures of the wild type and *glnG* mutant grown in morpholinepropanesulfonic acid (MOPS) minimal medium under high- or low-nitrogen conditions. We found that all NtrC-dependent genes were upregulated under nitrogen limitation in laboratory cultures (Fig. 2A). However, most NtrC activated genes and operons were downregulated in mice colonized with wild type, suggesting that nitrogen is not limiting in the mouse gut (Fig. 2A). Interestingly, the transcriptomes of the wild type and *glnG* mutant were highly similar, suggesting that induction of NtrC-dependent genes is not needed. Since NtrC is important for *E. coli* colonization (Fig. 1) despite the apparent abundance of nitrogenous compounds, this suggests that NtrC plays a role in fitness of colonized *E. coli* just as it does in laboratory cultures. Overall, these data indicate that nitrogen is not limiting for *E. coli* colonization.

### Amino acid biosynthetic pathways are downregulated in the mouse intestine

If amino acids are abundant in the mouse intestine, then bacteria can fill their amino acid pools and downregulate amino acid biosynthesis. In minimal medium, amino acid biosynthetic pathways are generally upregulated because bacteria need to generate these building blocks from glucose (27). We sought to determine if amino acid biosynthetic pathways are downregulated in colonized *E. coli* as an indicator of amino acid availability in the gut.

Amino acid biosynthetic pathways (Fig. S1) were downregulated in the mouse intestine (Fig. 2B) compared to cultures grown in MOPS minimal glucose medium, indicating the availability of amino acids. For example, genes involved in arginine and aspartic acid biosynthesis were significantly downregulated in the intestine. Aspartic acid serves as an intermediate in biosynthesis of lysine, methionine, asparagine, threonine, and isoleucine (28) and all genes encoding biosynthetic enzymes involved in their synthesis were downregulated. Also downregulated were genes involved in biosynthesis of glutamate and glutamine, the major intracellular nitrogen donors (8), which suggests there is abundant intracellular nitrogen in *E. coli*. All three genes involved in the biosynthesis of L-serine (29) were downregulated, as were genes involved in biosynthesis of glycine and cysteine. L-cysteine can be converted to L-alanine by desulfurase activity (*sufS* and *iscS*), depending on the cell’s requirement for sulfane sulfur (30). Although this pathway contributes minimally to the bacterial cell’s alanine requirement, *sufS* was upregulated in colonized *E. coli*, suggesting a requirement for sulfane sulfur in the intestine. Genes involved in alanine and proline biosynthesis also were modestly upregulated. Chorismate serves as an intermediate in the biosynthesis of tyrosine, tryptophan, and phenylalanine, and the first enzyme involved in chorismate biosynthesis, encoded by *aroF*, was downregulated, as were all genes involved in biosynthesis of aromatic amino acids. The *hisLGDCBHAFI* operon was downregulated; these gene products are involved in the biosynthesis of histidine and the common intermediate of leucine and valine synthesis. Downregulation of genes involved in amino acid biosynthesis is corroborated by previous findings that amino acid biosynthesis is repressed when *E. coli* is grown *in vitro* in cecal mucus (3).

### RNA-seq analysis reveals pathways used by colonized *E. coli* to assimilate nitrogen

**Fig 3:**
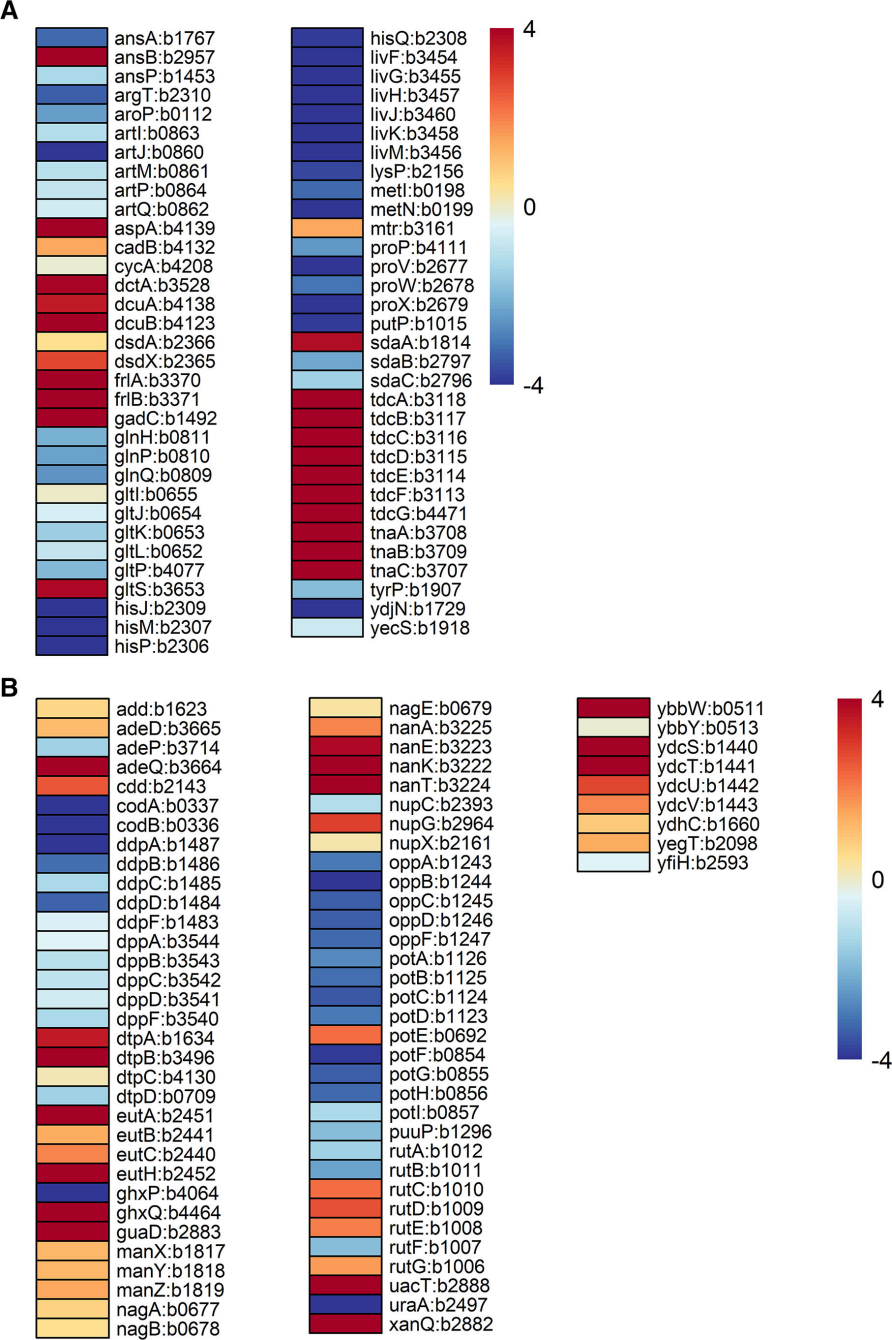
(A) Heatmap showing differential expression of genes involved in amino acid uptake, assimilation, or degradation in colonized *E. coli* MG1655 compared to *E. coli* MG1655 grown in MOPS minimal medium containing 0.2% glucose and 10 mM NH_4_Cl. (B) Heatmap showing differential expression of genes involved in uptake, assimilation, or degradation of nitrogenous compounds in colonized *E. coli* MG1655 compared to *E. coli* MG1655 grown in MOPS minimal medium containing 0.2% glucose and 10 mM NH_4_Cl.

To determine which pathways are used by *E. coli* to assimilate nitrogen in the intestine, we searched for upregulated nitrogen source uptake and degradation genes. We found that genes involved in the catabolism of seven different amino acids were upregulated (Fig. 3A). Asparagine permease encoded by *ansP* and cytoplasmic asparaginase I encoded by *ansA* were downregulated; however, periplasmic asparaginase II encoded by *ansB* (31) was highly upregulated. *E. coli* contains multiple transport systems for aspartate and glutamate: *gltIJKL* and *gltP*, which encode nonspecific permeases (32), were downregulated, whereas *dctA, dcuA*, and *dcuB* were upregulated in colonized *E. coli. aspA*, which encodes aspartase (aspartate ammonia-lyase), required for nitrogen assimilation from L-aspartate (10), was highly upregulated. Also upregulated in colonized *E. coli* were *gltS and gadC*, suggesting that glutamate could serve to protect *E. coli* from acid stress in the intestine. In this case, transport of glutamate via GadC does not result in net nitrogen gain, as the decarboxylated product γ-aminobutyric acid is exchanged for exogenous glutamate (33, 34). The periplasmic binding protein encoded by *argT*, together with an ABC transporter encoded by the *hisJQMP* operon, is involved in the transport of lysine. CadB, LysP, and fructoselysine transporter FrlA also transport lysine (35, 36). *cadB* and the *frlABCD* operon were upregulated in colonized *E. coli*, suggesting that lysine could serve to protect *E. coli* from acid stress in the intestine. CadB is a lysine-cadaverine antiporter which plays a role in pH homeostasis by importing lysine in exchange for the decarboxylated product cadaverine. So, like the Gad system, there is no net gain of nitrogen through the Cad system (35, 37). Fructoselysine, which is abundant in mouse chow as a product of nonenzymatic reaction of glucose with primary amines like lysine (38), could serve as a carbon and nitrogen source.

*E. coli* has at least five known L-serine transporters, encoded by *sdaC*, *tdcC*, *cycA*, *sstT*, and *dsdX* (39, 40) and three known L-serine deaminases, encoded by *sdaA*, *sdaB*, and *tdcG* (41). L-serine is important for *E. coli*, and deletion of all L-serine deaminases interferes with one-carbon metabolism and cell wall synthesis (42, 43) (Fig. 3A). Genes encoding the transporters TdcC and DsdX and deaminases SdaA and TdcG were upregulated in colonized *E. coli*. There are three known transport systems for threonine: SstT, TdcC, and common transport system LIV-I, and they also transport L-serine (44, 45). *E. coli* has multiple pathways to utilize threonine including deamination by threonine deaminase, encoded by *tdcB* (44)*.* All genes in the *tdcABCDEFG* operon were upregulated in colonized *E. coli*, which suggests that threonine could also be an important nutrient for *E. coli* in the intestine. Tryptophan is transported by three permeases: two tryptophan-specific transporters Mtr and TnaB and a general aromatic amino acid transporter, AroP (44, 46). *tnaA* encodes tryptophanase, which degrades tryptophan to indole, pyruvate, and ammonia (46). The *tnaABC* operon and *mtr* were upregulated in the mouse intestine, suggesting that *E. coli* also obtains tryptophan from the gut.

Next, we investigated other pathways involved in uptake and utilization of nitrogenous compounds, such as dipeptides, tripeptides and oligopeptides, purines and pyrimidines, urea, polyamines, and amino sugars (Fig. 3B). *ddpABCDF*, which encodes D-dipeptide uptake system to import D-alanyl-D-alanine (25), and *dppABCDF*, which encodes another dipeptide transporter (47), were downregulated in colonized *E. coli. E. coli* also has four peptide transporter family proteins: dipeptide and tripeptide permease A (DtpA), DtpB, DtpC, and DtpD; *dtpA* and *dtpB* were highly upregulated. *oppABCDF*, which supports catabolism of oligopeptides (48), was downregulated. Genes involved in the transport and/or utilization of urea (*uacT*), ethanolamine (*eut* operon), NANA (*nanATEK*), and glucosamine (*manXYZ*) were upregulated, as were genes involved in utilization of NAG (*nagE* and *nagBA*). The putative putrescine transporter pathway (*ydcSTUV*), predicted allantoin transporter (*ybbW*), and pathways involved in purine and pyrimidine utilization were also upregulated in colonized *E. coli* (Fig. 3B).

### L-serine, NANA, NAG, and di- and tripeptides are nitrogen sources for colonized *E. coli*

**Fig 4:**
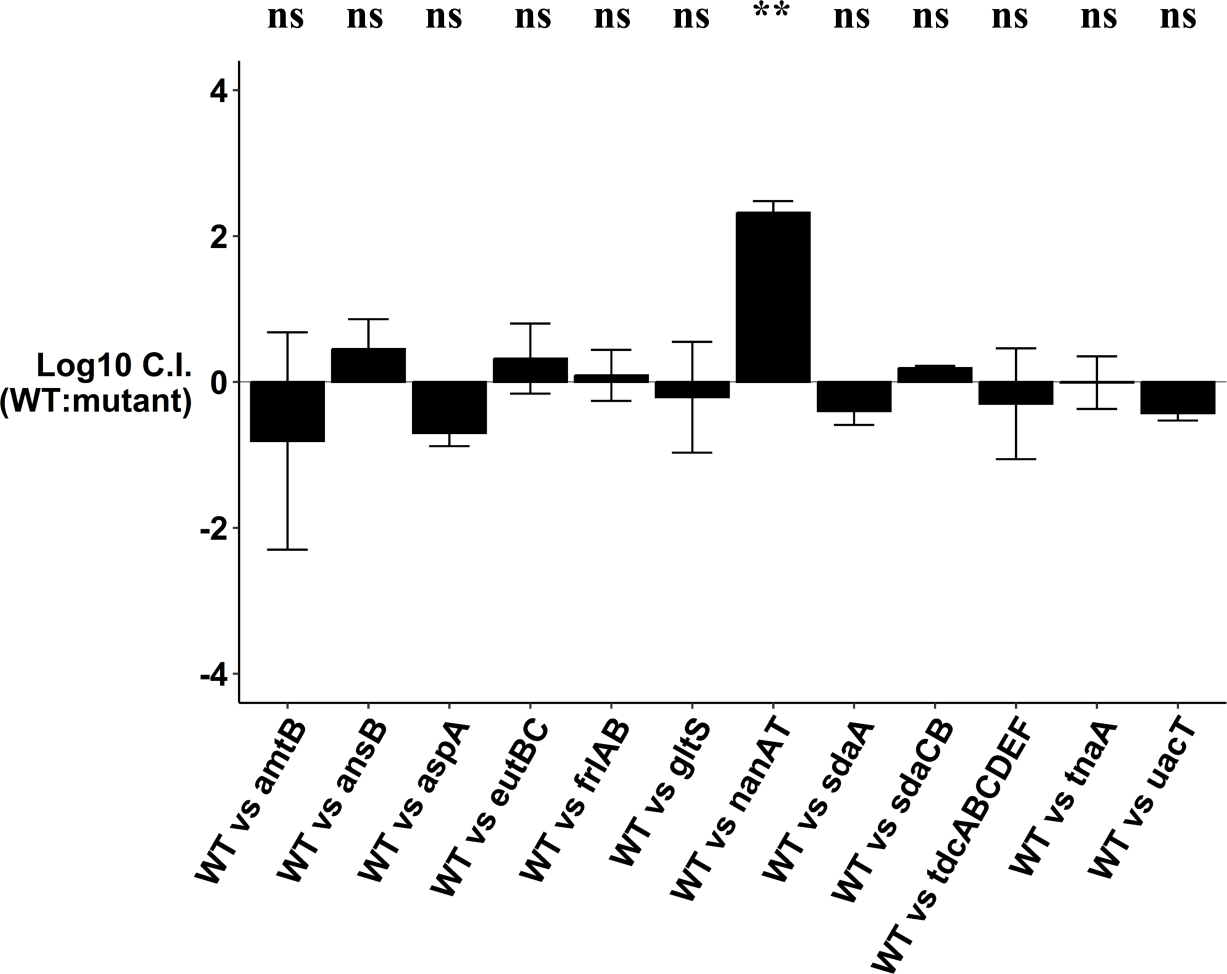
Log_10_ competitive index (wild type/mutant) at day 9 of competitive colonization of the mouse intestine by *E. coli* MG1655 Str^R^ Nal^R^ wild type and different single-knockout mutants. Bars represent the average competitive index. Error bars represent standard errors of the mean. **P < 0.01. ns, not statistically significant.

**Fig 5:**
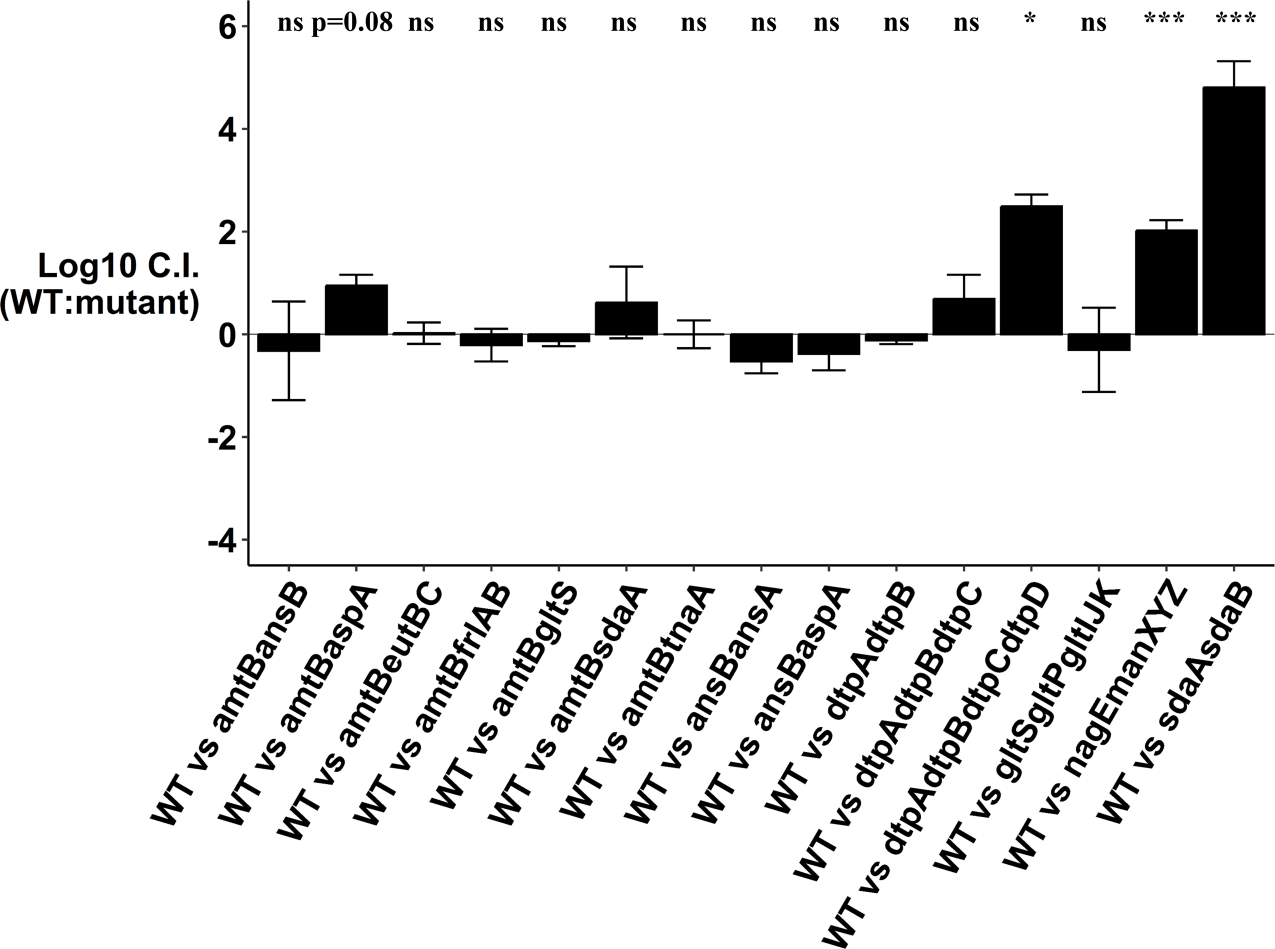
Log_10_ competitive index (wild type/mutant) at day 9 of competitive colonization of the mouse intestine by *E. coli* MG1655 Str^R^ Nal^R^ wild type and different double, triple, or quadruple knockout mutants. Bars represent the average competitive index. Error bars represent standard errors of the mean. *P < 0.05, ***P < 0.001. ns, not statistically significant.

To determine what nitrogenous compounds support *E. coli* colonization, we systematically knocked out transporters and/or enzymes involved in the degradation of these compounds and tested them for competitive colonization. A total of 12 single-knockout mutants and 15 double, triple, and quadruple mutants (Table 1) were assessed for competitive disadvantages in streptomycin-treated mice. When constructing the mutants, we took care to ensure the mutations were specific to the nutrient source in question and did not have growth defects. For instance, we did not use the *nagBA* deletion strain, which causes accumulation of toxic intermediates in the presence of NAG and results in a growth defect (3, 4). The log_10_ competitive index for the wild type vs single mutants at day 9 of competition is shown in Fig. 4, and the log_10_ competitive index for the wild type vs the double, triple, and quadruple mutants is shown in Fig. 5.

**TABLE 1:** *E. coli* strains and plasmids used in the study

*E. coli* strain	Description of the strain	Reference
*E. coli* MG1655 Str^R^	Spontaneous streptomycin-resistant mutant of MG1655	(49)
*E. coli* MG1655 Str^R^ Nal^R^	Spontaneous nalidixic acid-resistant mutant of MG1655 Str^R^	(49)
*E. coli* MG1655 Str^R^ ∆*glnG*::*cam*	*glnG* gene is deleted and replaced by a chloramphenicol resistance cassette.	This study
*E. coli* MG1655 Str^R^ ∆*amtB*:: *cam*	*amtB* gene is deleted and replaced by a chloramphenicol resistance cassette.	This study
*E. coli* MG1655 Str^R^ ∆*aspA*:: *cam*	*aspA* gene is deleted and replaced by a chloramphenicol resistance cassette.	This study
*E. coli* MG1655 Str^R^ ∆*ansB*::*cam*	*ansB* gene is deleted and replaced by a chloramphenicol resistance cassette.	This study
*E. coli* MG1655 Str^R^ ∆*ansB* ∆*ansA*::*cam*	*ansB ansA* double-deletion mutant of *E. coli* MG1655 Str^R^ carrying a chloramphenicol resistance cassette in the *ansA* deletion	This study
*E. coli* MG1655 Str^R^ ∆*frlAB*::*cam*	*frlA* and *frlB* genes in an operon are deleted and replaced by a chloramphenicol resistance cassette.	This study
*E. coli* MG1655 Str^R^ ∆*gltS::cam*	*gltS gene* is deleted and replaced by a chloramphenicol resistance cassette.	This study
*E. coli* MG1655 Str^R^ ∆*gltS* ∆*gltP* ∆*gltIJK::cam*	*gltS gltP gltIJK* triple deletion mutant of *E. coli* MG1655 Str^R^ carrying a chloramphenicol resistance cassette in the *gltIJK* deletion	This study
*E. coli* MG1655 Str^R^ ∆*sdaA::cam*	*sdaA* gene is deleted and replaced by a chloramphenicol resistance cassette.	This study
*E. coli* MG1655 Str^R^ ∆*sdaCB::cam*	*sdaC* and *sdaB* genes in an operon are deleted and replaced by a chloramphenicol resistance cassette.	This study
*E. coli* MG1655 Str^R^ ∆*sdaA*∆*sdaB::cam*	*sdaA* and *sdaB* double-deletion mutant of *E. coli* MG1655 Str^R^ carrying a chloramphenicol resistance cassette in the *sdaB* deletion	This study
*E. coli* MG1655 Str^R^ ∆*tdcABCDEF::cam*	Six genes in *tdc* operon were deleted and replaced by a chloramphenicol resistance cassette.	This study
*E. coli* MG1655 Str^R^ ∆*tnaA::cam*	*tnaA* gene is deleted and replaced by a chloramphenicol resistance cassette.	This study
*E. coli* MG1655 Str^R^ ∆*dtpA*∆*dtpB::cam*	*dtpA dtpB* double-deletion mutant of *E. coli* MG1655 Str^R^ carrying a chloramphenicol resistance cassette in the *dtpB* deletion	This study
*E. coli* MG1655 Str^R^ ∆*dtpA*∆*dtpB*∆*dtpC::cam*	*dtpA dtpB dtpC* triple deletion mutant of *E. coli* MG1655 Str^R^ carrying a chloramphenicol resistance cassette in the *dtpC* deletion	This study
*E. coli* MG1655 Str^R^ ∆*dtpA*∆*dtpB*∆*dtpC*∆*dtpD::cam*	*dtpA dtpB dtpC dtpD* quadraple deletion mutant of *E. coli* MG1655 Str^R^ carrying a chloramphenicol resistance cassette in the *dtpD* deletion	This study
*E. coli* MG1655 Str^R^ ∆*eutBC::cam*	*eutB* and *eutC* genes in *eut* cluster are deleted and replaced by chloramphenicol resistance cassette.	This study
*E. coli* MG1655 Str^R^ ∆*nagE*∆*manXYZ::kan*	*nagE manXYZ* double-deletion mutant of *E. coli* MG1655 Str^R^ carrying a kanamycin resistance cassette in the *manXYZ* deletion	(3)
*E. coli* MG1655 Str^R^ ∆*nanAT::kan*	*nanA* and *nanT* genes in an operon are deleted and replaced by kanamycin resistance cassette.	(3)
*E. coli* MG1655 Str^R^ ∆*uacT::cam*	uacT gene is deleted and replaced by chloramphenicol resistance cassette.	This study
*E. coli* MG1655 Str^R^*∆amtB∆ansB::cam*	*amtB ansB* double-deletion mutant of *E. coli* MG1655 Str^R^ carrying a chloramphenicol resistance cassette in the *ansB* deletion	This study
*E. coli* MG1655 Str^R^*∆amtB∆aspA::cam*	*amtB aspA* double-deletion mutant of *E. coli* MG1655 Str^R^ carrying a chloramphenicol resistance cassette in the *aspA* deletion	This study
*E. coli* MG1655 Str^R^ *∆amtB∆eutBC::cam*	*amtB eutBC* double-deletion mutant of *E. coli* MG1655 Str^R^ carrying a chloramphenicol resistance cassette in the *eutBC* deletion	This study
*E. coli* MG1655 Str^R^ *∆amtB∆frlAB::cam*	*amtB frlAB* double-deletion mutant of *E. coli* MG1655 Str^R^ carrying a chloramphenicol resistance cassette in the *frlAB* deletion	This study
*E. coli* MG1655 Str^R^ *∆amtB∆glnG::cam*	*amtB glnG* double-deletion mutant of *E. coli* MG1655 Str^R^ carrying a chloramphenicol resistance cassette in the *glnG* deletion	This study
*E. coli* MG1655 Str^R^ *∆amtB∆gltS::cam*	*amtB gltS* double-deletion mutant of *E. coli* MG1655 Str^R^ carrying a chloramphenicol resistance cassette in the *gltS* deletion	This study
*E. coli* MG1655 Str^R^ *∆amtB∆sdaA::cam*	*amtB sdaA* double-deletion mutant of *E. coli* MG1655 Str^R^ carrying a chloramphenicol resistance cassette in the *sdaA* deletion	This study
*E. coli* MG1655 Str^R^ *∆amtB∆tnaA::cam*	*amtB tnaA* double-deletion mutant of *E. coli* MG1655 Str^R^ carrying a chloramphenicol resistance cassette in the *tnaA* deletion	This study
*E. coli* MG1655 Str^R^ *∆ansB∆aspA::cam*	*ansB aspA* double-deletion mutant of *E. coli* MG1655 Str^R^ carrying a chloramphenicol resistance cassette in the *aspA* deletion	This study
pKD3	A template plasmid for *frt*-flanked chloramphenicol acetyltransferase cassette	(50)
pKD4	A template plasmid for *frt*-flanked kanamycin cassette	(50)
pCP20	A helper plasmid, which expresses the FLP recombinase	(50)
pSIJ8	The temperature-sensitive plasmid, which has a lambda red recombinase gene under the control of arabinose inducible promoter and flippase recombinase gene under the control of rhamnose inducible promoter	(51)

**Fig 6:**
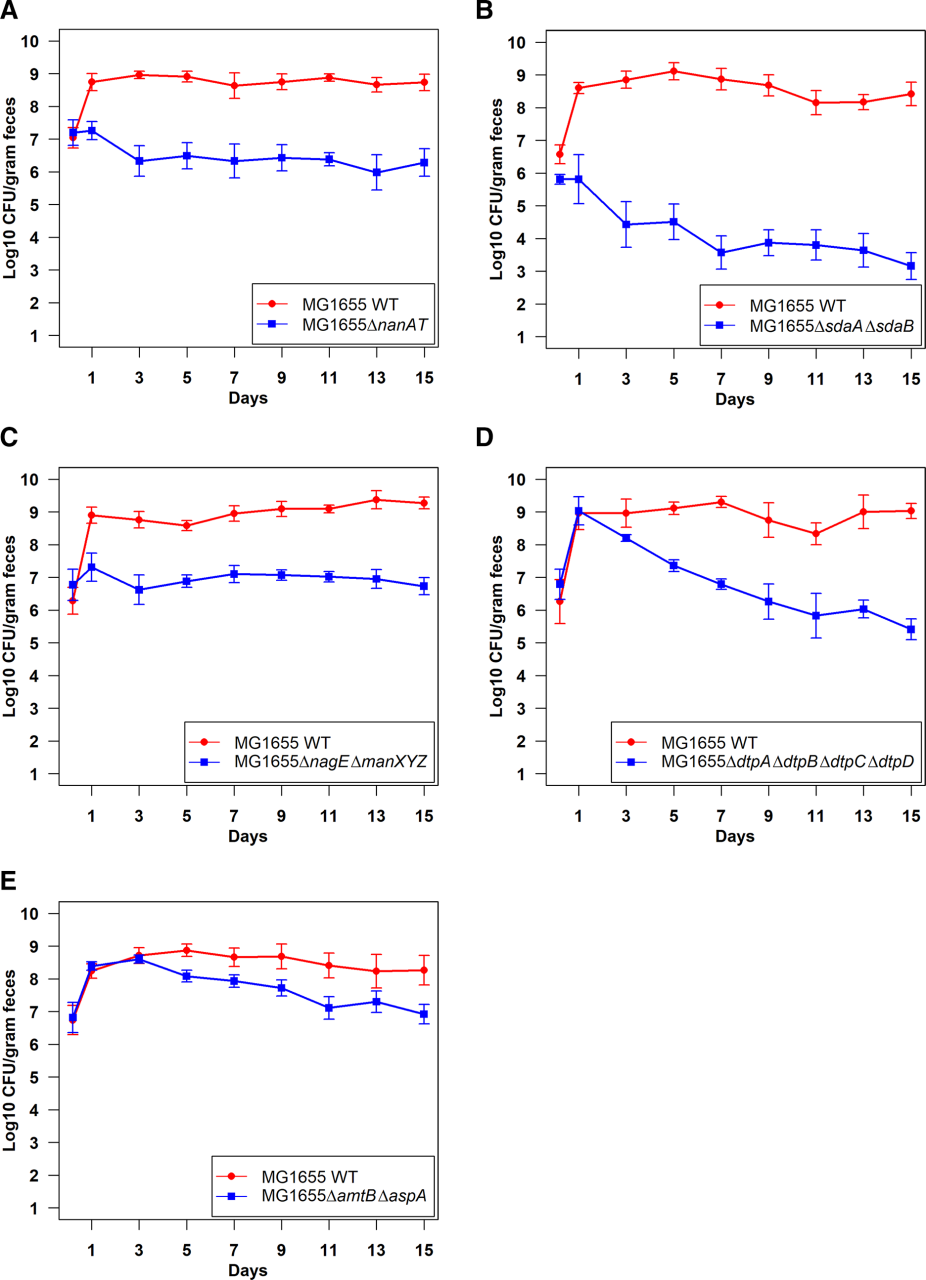
Mutants defective in competition against the wild type in the mouse intestine. (**A**) Competitive colonization of the mouse intestine by *E. coli* MG1655 Str^R^ Nal^R^ wild type and *E. coli* MG1655 Str^R^ ∆*nanAT::kan*.** (B)** Competitive colonization of the mouse intestine by *E. coli* MG1655 Str^R^ Nal^R^ wild type and *E. coli* MG1655 Str^R^ ∆*sdaA* ∆*sdaB::cam*.** (C)** Competitive colonization of the mouse intestine by *E. coli* MG1655 Str^R^ Nal^R^ wild type and *E. coli* MG1655 Str^R^ ∆*nagE* ∆*manXYZ::kan*.** (D)** Competitive colonization of the mouse intestine by *E. coli* MG1655 Str^R^ Nal^R^ wild type and *E. coli* MG1655 Str^R^ ∆*dtpA* ∆*dtpB* ∆*dtpC* ∆*dtpD::cam*. (**E)** Competitive colonization of the mouse intestine by *E. coli* MG1655 Str^R^ Nal^R^ wild type and *E. coli* MG1655 Str^R^ ∆*amtB* ∆*aspA::cam*.

Although ammonia is present in the intestine of mammals (24, 52) and is a preferred nitrogen source that supports fastest growth of *E. coli* (8), we found the ammonia transporter (*amtB*) was not upregulated in the mouse intestine (Fig. 2). The *amtB* mutant did not have a colonization defect when competing against the wild type (Fig. S2), suggesting that active ammonia transport is not necessary for *E. coli* to colonize. However, ammonia can diffuse across the cytoplasmic membrane (53). We grew the wild type and *amtB* mutant in MOPS minimal medium containing high (10 mM NH_4_Cl) and low nitrogen (3 mM NH_4_Cl), and both strains grew equally well (Fig. S3), supporting previous evidence that the ammonia transporter is needed to sustain growth only if the ammonium concentration is below 20 µM at neutral pH (53) or below 1 mM at pH 5(54). These results do not rule out the possibility that ammonia supplies nitrogen to colonized *E. coli.*

Single knockouts eliminating uptake or degradation of asparagine*,* aspartate*,* ethanolamine*,* fructoselysine*,* glutamate*,* L-serine*,* threonine*,* tryptophan, and urea did not show colonization defects (Fig. 4; Fig. S4). Mutation deleting genes involved in degradation of asparagine and another blocking transport of glutamate colonized equally well as the wild type, indicating they are not nitrogen sources for *E. coli* (Fig. 5; Fig. S5). However, a NANA utilization mutant was defective in competition with the wild type (Fig. 4 and 6A). This supports our previous findings (3, 4) that NANA is a nutrient used by *E. coli* to colonize.

The single-deletion mutants (∆*sdaA* and ∆*sdaCB*) competed equally well against wild type (Fig. 4; Fig. S4). However, an L-serine deaminase double mutant was outcompeted by nearly 5 log_10_-fold by the wild type, which indicates L-serine is used by *E. coli* to colonize (Fig. 5 and 6B). When the L-serine deaminase double mutant was fed alone to streptomycin-treated mice, it colonized well (Fig. S6)**.** The mutant did not have a growth defect *in vitro*, as it grew equally well as the wild type in lysogeny broth (LB), LB + 0.2% glucose, and LB + 0.2% phosphoenolpyruvate (Fig. S6). Although three L-L-serine deaminases are present in *E. coli* MG1655, we did not construct the triple deletion as the complete inability of *E. coli* K-12 to deaminate L-serine impairs growth and cell division (41).

A double mutant with deletions in NAG uptake (∆*nagE* ∆*manXYZ*) was also defective in competition against the wild type (Fig. 5 and 6C), which corroborates previous evidence (3, 4) that NAG supports growth of *E. coli* in the intestine. We also constructed mutants for the dipeptide and tripeptide permeases (∆*dtpA* ∆*dtpB*, ∆*dtpA* ∆*dtpB* ∆*dtpC*, and ∆*dtpA* ∆*dtpB* ∆*dtpC* ∆*dtpD*) and assessed their competitive fitness against the wild type in mice. The double and triple mutants colonized equally well as the wild type (Fig. 5; Fig. S5). However, the quadruple mutant had a significant defect by day 9 of competition (Fig. 5 and 6D). This suggests that uptake of di- and tripeptides is important for *E. coli* colonization.

To test the hypothesis that a nitrogen source which is not limiting individually may become limiting when combined with another defect, we constructed some mutants with deletions in two different functions. These mutants were mostly constructed in the ammonia transporter mutant background since ammonia is a preferred nitrogen source *in vitro* and is available in the intestine. Out of eight double mutants with deletions in two different functions, seven did not have a defect in competitive colonization (Fig. 5; Fig. S5)**.** By contrast, the ∆*amtB* ∆*aspA* mutant was modestly outcompeted by the wild type by day 15 of competition (*P* = 0.04) (Fig. 5 and 6E). This suggests that aspartate and ammonia, although not limiting individually, together contribute modestly as nitrogen sources for *E. coli* in the intestine. Overall, these experiments demonstrate that L-serine, NANA, NAG, di- and tripeptides, aspartate, and ammonia are important nitrogen sources used by *E. coli* to colonize the mammalian intestine.

### Confirmation that L-serine and NANA are important nitrogen sources for *E. coli* in the intestine

L-serine, NAG, and NANA could, theoretically, serve both as carbon and nitrogen sources. L-serine deaminases produce pyruvate and ammonia (55), and pyruvate is a substrate for gluconeogenesis and the tricarboxylic acid (TCA) cycle, while ammonia is the preferred nitrogen source (14). However, L-serine cannot serve as a sole source of carbon *in vitro*, although it can function as sole nitrogen source, indicating its potential as a nitrogen source rather than a carbon source (44, 56–58). NAG is deacetylated by NagA and deaminated by NagB, resulting in production of fructose-6-P (59), which enters glycolysis. The ammonia generated from NAG can supply the nitrogen requirement. NANA is also converted to NAG by NanA, NanK and NanE enzymes, with production of pyruvate (59).

Thus, we sought to determine whether *E. coli* utilizes L-serine, NAG, or NANA as carbon or nitrogen sources or both. We designed a strategy to assess the competitive fitness of *E. coli* wild type and mutants in mice with an excess of a readily metabolizable carbon source (gluconate) or nitrogen source (NH_4_Cl) or both. We reasoned that a continuous supply of these compounds in the drinking water would rescue mutants with carbon or nitrogen utilization deficiencies. This experimental approach was successfully employed in our previous study in which *E. coli* EDL933 and *E. coli* MG1655 *glgP* mutants unable to degrade glycogen and defective in competition with their parent strains were rescued by supplying gluconate in the drinking water during competitive colonization (60).

**Fig 7:**
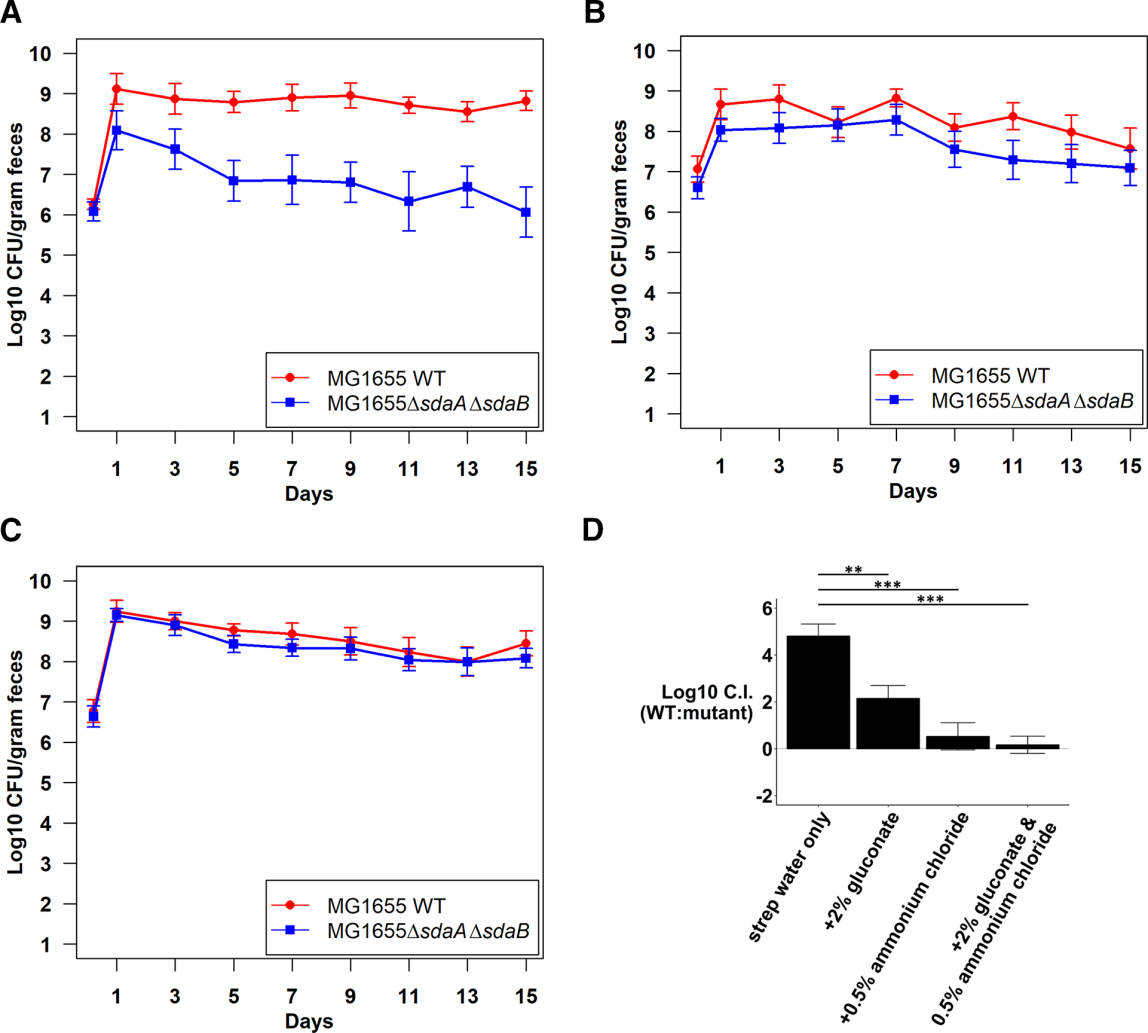
Competitive colonization of the mouse intestine by *E. coli* MG1655 Str^R^ Nal^R^ wild type and *E. coli* MG1655 Str^R^ ∆*sdaA* ∆*sdaB::cam* under different conditions.** (A)** Competitive colonization in mice fed streptomycin water supplemented with 2% gluconate. (B) Competitive colonization in mice fed streptomycin water supplemented with 0.5% NH_4_Cl. (**C)** Competitive colonization in mice fed streptomycin water supplemented with 2% gluconate and 0.5% NH_4_Cl. (**D)** Log_10_ competitive index (wild type/∆*sdaA* ∆*sdaB*) at day 9 of competitive colonization under different conditions (two tailed *t*-test assuming equal variances shows statistical significance). **P < 0.01, ***P < 0.001.

Drinking water containing 2% gluconate partially rescued the competition defect of the ∆*sdaA* ∆*sdaB* mutant in competition against the wild type (compare Fig. 6 and 7A). However, a continuous supply of 0.5% NH_4_Cl in the drinking water almost completely rescued the mutant (Fig. 7B). As anticipated, the addition of gluconate plus NH_4_Cl in the drinking water completely rescued the mutant (Fig. 7C). The log_10_ competitive indices on day 9 of these competitions are shown in Fig. 7D. The results confirm that L-serine serves primarily as a nitrogen source.

**Fig 8:**
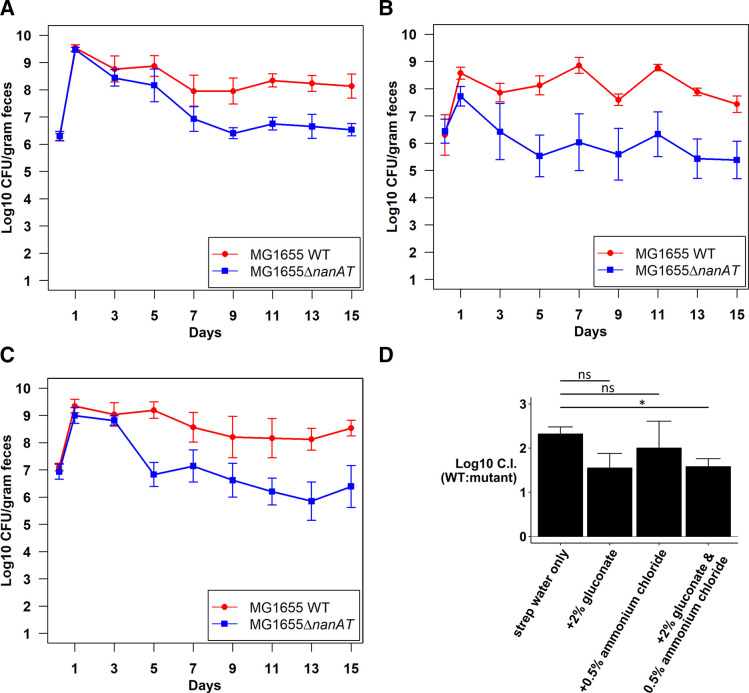
Competitive colonization of the mouse intestine by *E. coli* MG1655 Str^R^ Nal^R^ wild type and *E. coli* MG1655 Str^R^ ∆*nanAT::kan* under different conditions.** (A)** Competitive colonization in mice fed streptomycin water supplemented with 2% gluconate. (B) Competitive colonization in mice fed streptomycin water supplemented with 0.5% NH_4_Cl. (**C)** Competitive colonization in mice fed streptomycin water supplemented with 2% gluconate and 0.5% NH_4_Cl. (**D)** Log_10_ competitive index (wild type/∆*nanAT*) at day 9 of competitive colonization under different conditions. ns indicates *P* value ≥0.05. *P < 0.05.

To determine whether NANA is a nitrogen or carbon source or both, we assessed the competitive fitness of the ∆*nanAT* mutant against the wild type with supplemented drinking water. The competitive defect of the ∆*nanAT* mutant was not rescued by either gluconate or ammonia alone and was only partially rescued by gluconate plus ammonia (compare Fig. 6 and 8). This result suggests an additional role for NANA, besides minimally contributing as a carbon and nitrogen source for *E. coli* in the intestine.

**Fig 9:**
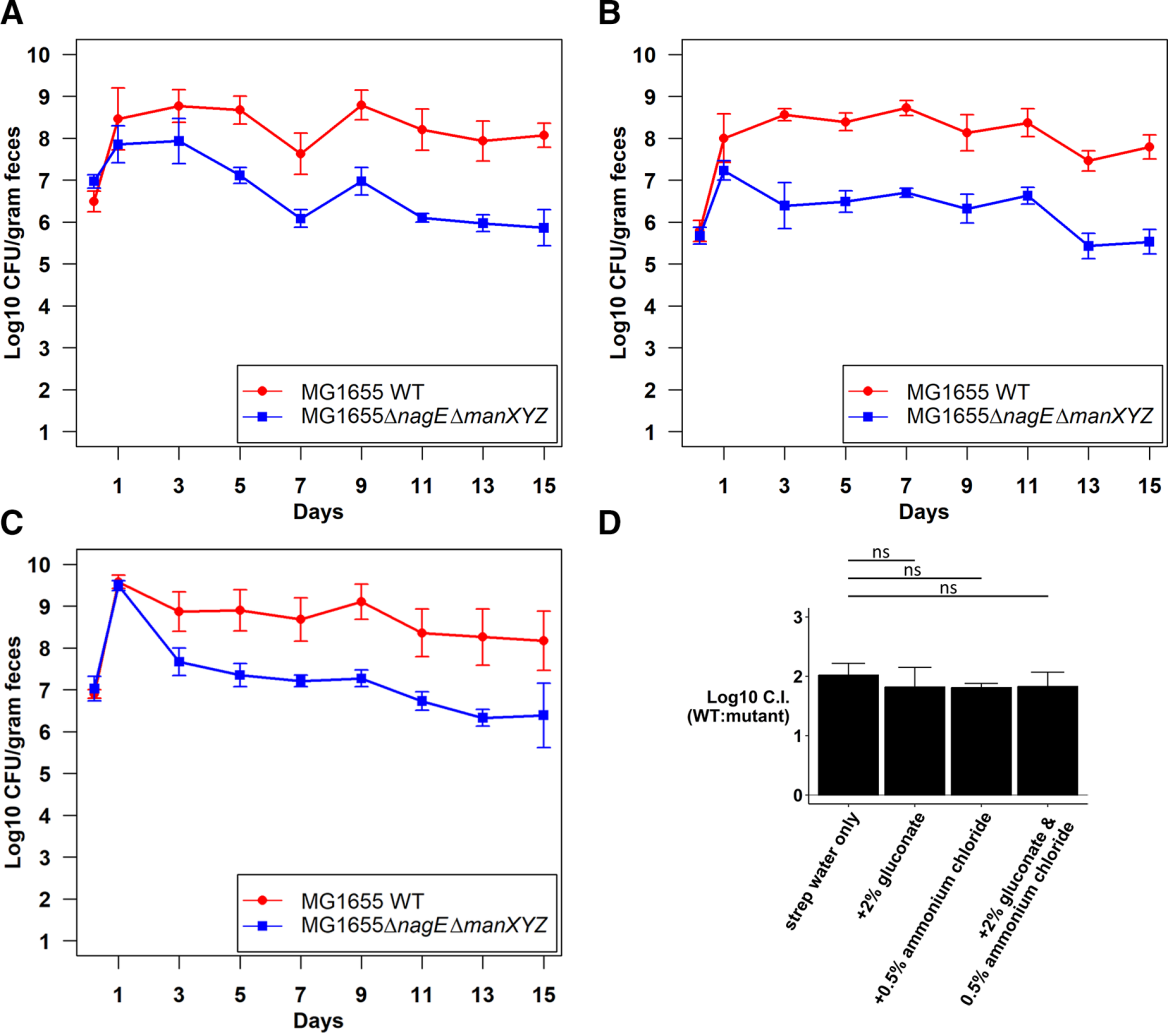
Competitive colonization of the mouse intestine by *E. coli* MG1655 Str^R^ Nal^R^ wild type and *E. coli* MG1655 Str^R^ ∆*nagE* ∆*manXYZ::kan* under different conditions.** (A)** Competitive colonization in mice fed streptomycin water supplemented with 2% gluconate. (B) Competitive colonization in mice fed streptomycin water supplemented with 0.5% NH_4_Cl. (**C) **Competitive colonization in mice fed streptomycin water supplemented with 2% gluconate and 0.5% NH_4_Cl. (**D)** Log_10_ competitive index (WT: ∆*nagE* ∆*manXYZ*) at day 9 of competitive colonization of the mouse intestine under different conditions (ns indicates *P* value ≥ 0.05).

We next determined whether NAG is a nitrogen or carbon source or both by competing the ∆*nagE* ∆*manXYZ* mutant against the wild type with supplemented drinking water (compare Fig. 6 and 9). Neither gluconate nor NH_4_Cl alone nor in combination could rescue the colonization defect. The log_10_ competitive indices on day 9 of competition is shown in Fig. 9D. Together these results suggest that NANA and NAG have a primary function other than serving as a carbon or nitrogen source, perhaps as a source of precursors for peptidoglycan biosynthesis.

## DISCUSSION

In this study, we found that a *glnG* mutant had a competitive defect in the intestine compared to its isogenic *E. coli* wild type. However, when the *glnG* mutant was fed alone to mice, it colonized at high numbers. Moreover, genes regulated by NtrC to alleviate nitrogen limitation were not upregulated in the mouse intestine. One interpretation of these results is that the previously noted fitness defect of a *glnG* mutant is responsible for its colonization defect. We previously determined that mutants lacking other global regulators, including aerobic respiratory control or fumarate nitrate reductase control, colonized at high numbers when fed alone but had substantial colonization defects in competition with their wild-type parent strains (61). Thus, none of these three global regulators is necessary for colonization, yet loss of any one of them results in a competitive fitness defect, perhaps due to the loss of coordinated gene expression and integration of metabolic signals.

Amino acid biosynthetic pathways were downregulated in colonized mice, indicating an abundance of nitrogenous compounds. RNA-seq analysis of *E. coli* isolated from the mouse cecum revealed upregulation of several genes, operons, and regulons involved in the utilization of nitrogenous compounds. Through systematic testing of knockout mutants, we demonstrated that L-serine, NANA, NAG, and di- and tripeptide support competitive colonization. Rescue experiments established that L-serine is used primarily as a nitrogen source, whereas NANA serves as a carbon and nitrogen source. Additionally, NAG and NANA appear to have functions other than serving as carbon or nitrogen sources, such as precursors for peptidoglycan biosynthesis.

Genes encoding pathways for utilization of asparagine, aspartate, glutamate, lysine, L-serine, threonine, and tryptophan were upregulated in the mouse intestine, suggesting these amino acids are important for *E. coli* colonization. However, competitive colonization experiments showed that L-serine is the only amino acid limiting the growth of *E. coli* in the mouse intestine. Aspartate alone does not appear to be limiting, corroborating previous findings (3, 11). However, when the ammonia transporter and aspartase were deleted, the double mutant had a minor, although significant, colonization defect, suggesting modest use of aspartate in the intestine. The L-serine deaminase mutant had the largest colonization defect of all mutants tested, indicating L-serine is the primary nitrogen source for *E. coli* in the intestine.

When grown in tryptone broth *in vitro*, *E. coli* consumes L-serine first, followed sequentially by aspartate and tryptophan, and then glutamate, glycine, threonine, and alanine (62). Selvarasu et al. (63) reported a similar order of amino acid consumption in complex medium: L-serine was consumed first, followed by aspartate and glutamate. Recently, it was shown that dietary L-serine confers an advantage to pathogenic *E. coli* infecting the mouse intestine (14). The gel-forming mucin, which is the major component of the colonic mucus, is a glycoprotein with a central protein core abundant in proline, threonine, and L-serine (64–66). Besides glycoproteins, phosphatidylserine can serve as both a carbon and nitrogen source for *E. coli* (67). Despite its apparent importance for colonization, L-serine cannot serve as a sole source of carbon, although it can function as a sole nitrogen source (44, 56–58). In laboratory cultures grown on minimal media, glucose plus L-serine supports a growth rate equal to that of glucose plus all 20 amino acids and grows significantly faster compared to minimal glucose without L-serine (68). Thus, L-serine is readily available to *E. coli* in the mucus layer and supports rapid growth when sugars are available.

Pathways for utilizing NAG, NANA, dipeptides, and tripeptides were also upregulated in the mouse intestine. The corresponding mutants were defective in colonization. These findings support previous evidence that NANA and NAG are important nutrients for *E. coli* in the intestine (3). NAG and NANA are commonly found in the glycan chains of the mucin (69). Anaerobes produce glycolytic enzymes to degrade host as well as dietary glycans, and *E. coli* utilizes monosaccharides such as NANA and NAG liberated during the hydrolysis of those complex polysaccharides (4, 70). *E. coli* contains four different peptide transporters (71), and deletion of two or three of the transporter genes did not affect colonization, whereas deletion of all four genes conferred a significant colonization defect. Di- and tripeptides are known to provide amino acids needed for protein synthesis (72).

Confirmation that L-serine, NANA, and NAG serve as nitrogen and/or carbon and energy sources was demonstrated in experiments designed to rescue the corresponding mutants with excess carbon (2% gluconate) or nitrogen (0.5% NH_4_Cl) in the drinking water during competitive colonization experiments. The results demonstrated that L-serine acts primarily as a nitrogen source for *E. coli* in the intestine and is not a major carbon source. This finding is consistent with previous studies showing that *E. coli* cannot use L-serine as a sole carbon source but can use it as an additional carbon source when other carbon sources are present (56, 57). Moreover, growth of *E. coli* is fastest on sugars when L-serine is present (68). Selvarasu et al. (63) showed that much of the pyruvate derived from L-serine is subsequently excreted from the cell. A *nanAT* mutant was partially rescued by excess carbon, suggesting that NANA contributes carbon and energy for *E. coli* in the intestine. Interestingly, a *nagE manXYZ* mutant was not rescued by either excess carbon or nitrogen, suggesting that NAG has an additional role in *E. coli* in the intestine. NANA is converted to NAG with the release of pyruvate, which could provide carbon and energy for *E. coli* in the intestine. It is possible that NAG, directly taken up from the gut or generated from breakdown of NANA, is primarily channeled to precursors of peptidoglycan. In conclusion, our findings demonstrate for the first time that L-serine is an important nitrogen source for *E. coli* in the mammalian intestine.

## MATERIALS AND METHODS

### Bacterial strains and plasmids

The bacterial strains and plasmids used in this study are listed in Table 1. The sequenced *E. coli* MG1655 strain was derived from its K-12 parent by curing bacteriophage lambda by means of UV light and F plasmid by acridine orange treatment (73). *E. coli* MG1655 Str^R^ (streptomycin resistant) and *E. coli* MG1655 Str^R^ Nal^R^ (nalidixic acid resistant) were used throughout this study (49).

### Testing for growth defects

*E. coli* strains were grown overnight in LB broth supplemented with 100 µg/mL of streptomycin sulfate at 37°C with shaking. The overnight cultures were then diluted 1:1,000 in LB, with or without 0.2% glucose or 0.2% phosphoenolpyruvate. Subsequently, 150 µL of diluted culture was pipetted into 96-well microtiter plates, and growth at 37°C was monitored spectrophotometrically using a microplate reader (Bio Tek Synergy H1). The experiment was repeated in triplicate.

### Growth of strains in high- and low-nitrogen minimal medium

*E. coli* strains were grown overnight in MOPS minimal medium (74) containing 0.05% glucose with shaking at 37°C. The overnight culture was then diluted to optical density of approximately 0.01 in 50 mL of high nitrogen (10 mM NH_4_Cl) or low nitrogen (3 mM) MOPS medium containing 0.2% glucose. The cultures were grown at 37°C with shaking at 200 RPM. Absorbance was measured at 600 nm using an Eppendorf BioPhotometer. The experiments were duplicated.

### Construction of mutants

*E. coli* MG1655 Str^R^ ∆*glnG::cam* was constructed through P1 phage transduction from donor strain (BW38028 ∆*glnG::cam*) as described previously (75). For all other deletion mutants, the allelic exchange method was used as described previously by Datsenko and Wanner (50), with modifications developed by Jensen et al. (51). For construction of multiple gene deletions, the first antibiotic resistance cassette was removed with FLP recombinase. Additional deletions were accomplished by sequential allelic replacement and confirmed by PCR using primers specific to the gene and cassette sequences.

### Mouse colonization experiments

Mouse colonization experiments were performed as described previously (49, 73, 76–79). Briefly, at least two sets of three CD-1 male mice (Charles River Laboratories, Wilmington, MA), aged 6–8 weeks, were acclimatized for 5 days, then transferred to individual cages and provided water containing streptomycin sulfate (5 g/L) for 24 h. Streptomycin sulfate selectively eliminates resident facultative anaerobes, which opens a niche for introduced *E. coli*. Next, the mice were starved for food and water for 18 h, and then fed 10^5^ CFU of one or more *E. coli* strains in 1 mL of 5% sucrose solution (day 0). Thereafter, food and streptomycin water were provided *ad libitum*. Fecal samples were collected at 5 and 24 h and odd-numbered days for 15 days. The fecal samples were homogenized, serially diluted, and plated on MacConkey agar plates containing appropriate antibiotics. The log_10_ CFU/g of feces was determined for each time point, strain, and mouse, and standard errors were calculated. Data from at least two independent experiments were pooled (≥6 mice total). A competitive index of ≥10^1^ was consistently significant (*P* < 0.05).

For rescue experiments, the mice were fed drinking water containing streptomycin sulfate (5 g/L) supplemented with either 2% gluconate, 0.5% NH_4_Cl, or both. The drinking water was made available *ad libitum*.

### Extraction and sequencing of bacterial RNA from mouse cecal mucus and *in vitro* cultures

Streptomycin-treated mice were colonized separately with *E. coli* MG1655 Str^R^ Nal^R^ wild type or *E. coli* MG1655 Str^R^ ∆*glnG::cam*, and on day 9 of colonization, when the mice were stably colonized, the mice were euthanized and total RNA was extracted from cecal mucus as previously described (79). Briefly, the cecal contents were squeezed out, and the lumen of the ceca was rinsed with Hanks salt solution (Thermo Scientific, catalog no. 88284). Subsequently, the cecal mucus was dislodged into 500 µL of Hanks salt solution, and this solution was transferred to a Lysis Matrix B tube (MP Biochemicals, catalog no. 116911100) containing 500 µL of phenol-chloroform-isoamyl alcohol (PCA at 25:24:1) and 210 µL of 20% SDS. The cells were lysed by bead beating for 70 s using a Bead Bug microtube compact homogenizer at 4,000 rpm (Benchmark Scientific, model no. 1030E). The aqueous phase was collected by centrifugation at 4°C and then transferred to a Phase Lock Gel tube (Quantabio, Beverly, MA; catalog no. 2302830) containing 500 µL of PCA, mixed, and centrifuged again. The sample was washed twice with 250-µL chloroform, mixed with sodium acetate and isopropanol, and stored at −70°C. Tubes were thawed and centrifuged, and the pellet was washed with 70% ethanol and air-dried. The dried pellet was dissolved in RNase/DNase-free water and quantified. The RNA samples were treated with DNase I to deplete DNA (Invitrogen, catalog no. AM2238).

Cultures grown in MOPS minimal medium were harvested at *A*_600_ of ~0.4–0.6 for exponential phase bacteria by mixing 5 mL of culture with 10 mL of RNA-protect reagent (Qiagen, catalog no. 76506). After 5 min, the cells were pelleted, and RNA was extracted using a RNeasy mini kit (Qiagen, catalog no. 74104) following the manufacturer’s instructions. Stationary-phase samples were obtained 30 min after the culture stopped growing. RNA samples were prepared in duplicate. The approach used for RNA sequencing and data analysis was described previously (79).

## Data Availability

The RNA-seq data are available at Gene Expression Omnibus (GEO) under accession no. GSE217743 and GSE241658.

## SUPPLEMENTAL MATERIAL

Supplemental material mbio.00025-24-s0001.pdf Supplemental figures.

ASM does not own the copyrights to Supplemental Material that may be linked to, or accessed through, an article. The authors have granted ASM a non-exclusive, world-wide license to publish the Supplemental Material files. Please contact the corresponding author directly for reuse.
