# Supplementary material for: Nitrogen assimilation by E. coli in the mammalian intestine
Source: mBio. 2024 Feb 21;15(3):e00025-24. doi: 10.1128/mbio.00025-24 (PMC10936423; doi:10.1128/mbio.00025-24)
Supplement: Supplemental material — Supplemental figures. [file mbio.00025-24-s0001.pdf]

Supplementary information for

E. coli utilizes L-serine as a major nitrogen source to colonize the mammalian intestine

Sudhir Doranga and Tyrrell Conway*
Department of Microbiology and Molecular Genetics, Oklahoma State University, Stillwater, OK 74078

*Corresponding Author:
Tyrrell Conway
Phone: 405-744-6243
Fax: 405-744-6790
e-mail: tconway@okstate.edu

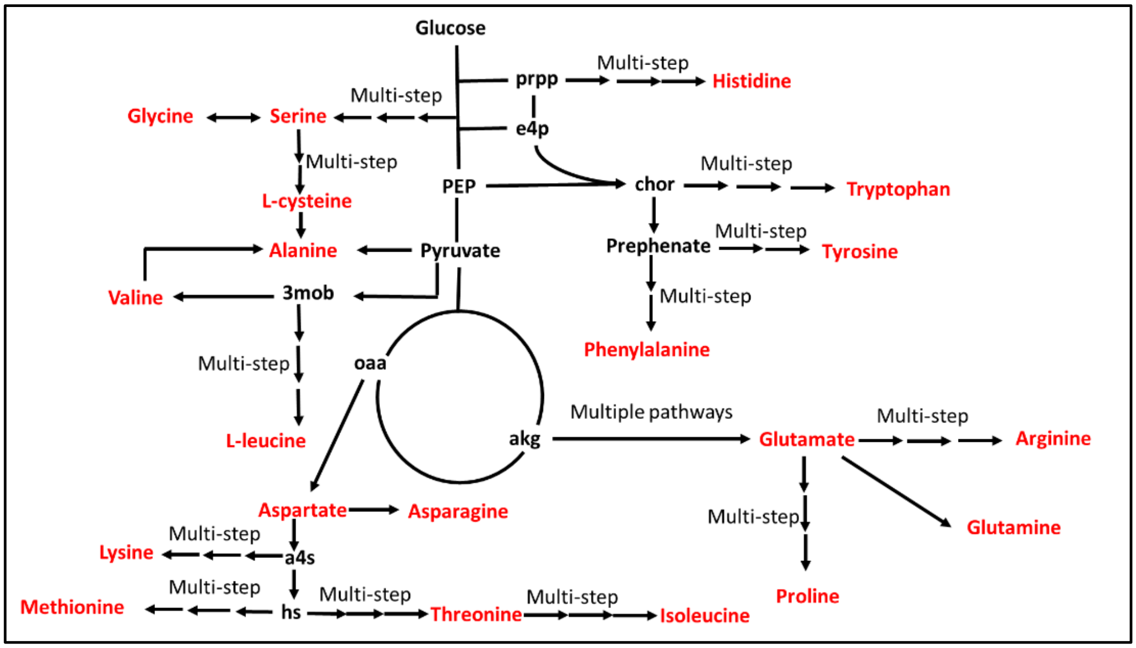

Figure S1. **Amino acid de novo synthesis from intermediates of glycolysis and TCA cycle**

(Adapted from Sander *et al*., (1)and Keseler *et al*.,(2))

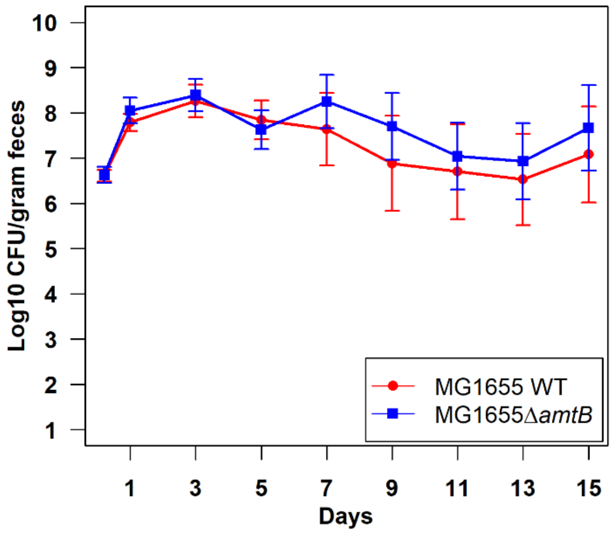

Figure S2. Colonization of the mouse intestine by *E. coli* MG1655 Str^R Nal^R Wild Type and *E. coli* MG1655 Str^R Δ*amtB::cam* mutant. Two sets of three CD-1 male mice were fed 10^5 CFU

of *E. coli* MG1655 Str^R Nal^R Wild Type and 10^5 CFU of *E. coli* MG1655 Str^R Δ*amtB::cam*. At indicated times, samples were collected, homogenized, diluted, and plated. Error bars represent standard errors of the pooled data of log_{10} mean CFU per gram of feces.

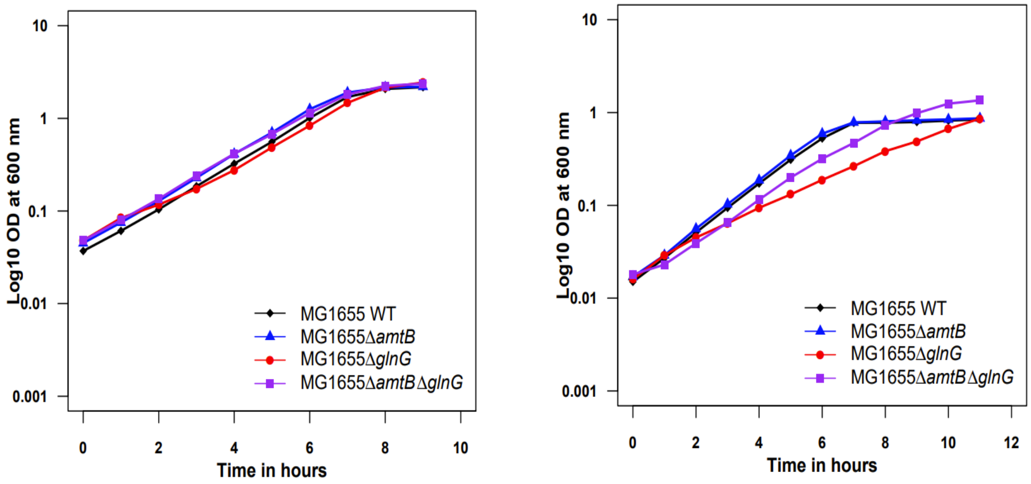

Figure S3. Growth of the *E. coli* MG1655 Str^R Nal^R wild type, *E. coli* MG1655 Str^R Δ*amtB*::*cam,* *E. coli* MG1655 Str^R Δ*glnG*::*cam* and *E. coli* MG1655 Str^R Δ*amtB* Δ*glnG::cam* mutants in MOPS medium containing different concentration of NH_4Cl. **A**. Growth of *E. coli* strains in MOPS medium containing 10 mM NH_4Cl; **B.** growth of *E. coli* strains in MOPS medium containing 3 mM NH_4Cl.

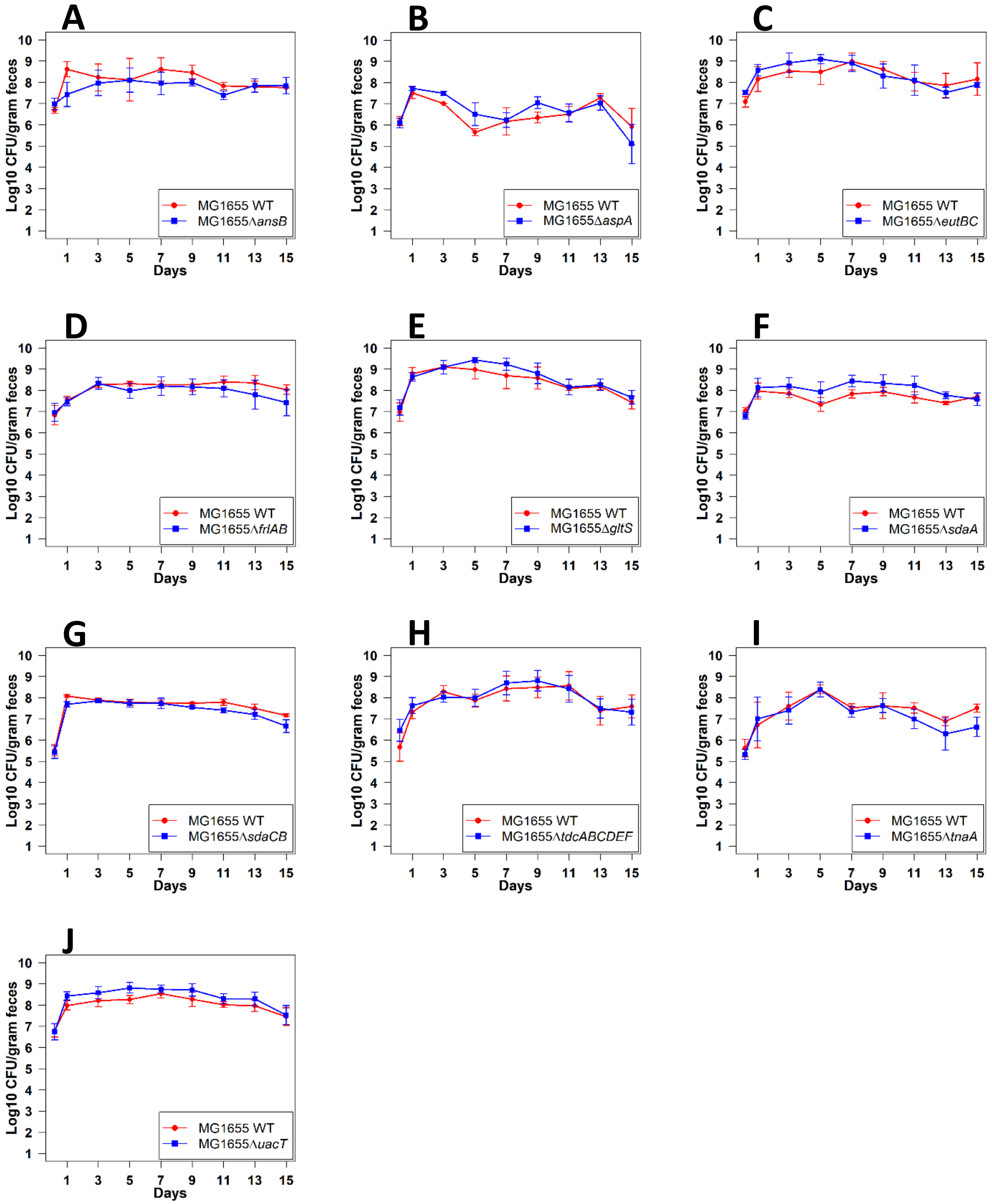

Figure S4. Competitive colonization of the mouse intestine by *E. coli* MG1655 Str^R Nal^R Wild Type and a single deletion mutant. One or two sets of three CD-1 male mice were fed

10^5 CFU of *E. coli* MG1655 Str^R Nal^R Wild Type and 10^5 CFU of *E. coli* mutant. At indicated times, samples were collected, homogenized, diluted, and plated. Error bars represent standard errors of the pooled data of $\log_{10}$ mean CFU per gram of feces. **A.** competition between *E. coli* MG1655 Str^R Nal^R Wild Type and *E. coli* MG1655 Str^R Δ*ansB*::*cam* **B.** competition between *E. coli* MG1655 Str^R Nal^R Wild Type and *E. coli* MG1655 Str^R Δ*aspA*::*cam* **C.** competition between *E. coli* MG1655 Str^R Nal^R Wild Type and *E. coli* MG1655 Str^R Δ*eutBC*::*cam* **D.** competition between *E. coli* MG1655 Str^R Nal^R Wild Type and *E. coli* MG1655 Str^R Δ*frlAB*::*cam* **E.** competition between *E. coli* MG1655 Str^R Nal^R Wild Type and *E. coli* MG1655 Str^R Δ*gltS*::*cam* **F.** competition between *E. coli* MG1655 Str^R Nal^R Wild Type and *E. coli* MG1655 Str^R Δ*sdaA*::*cam* **G.** competition between *E. coli* MG1655 Str^R Nal^R Wild Type and *E. coli* MG1655 Str^R Δ*sdaCB*::*cam.* **H.** competition between *E. coli* MG1655 Str^R Nal^R Wild Type and *E. coli* MG1655 Str^R Δ*tdcABCDEF*::*cam* **I.** competition between *E. coli* MG1655 Str^R Nal^R Wild Type and *E. coli* MG1655 Str^R Δ*tnaA*::*cam* **J.** competition between *E. coli* MG1655 Str^R Nal^R Wild Type and *E. coli* MG1655 Str^R Δ*uacT*::*cam.*

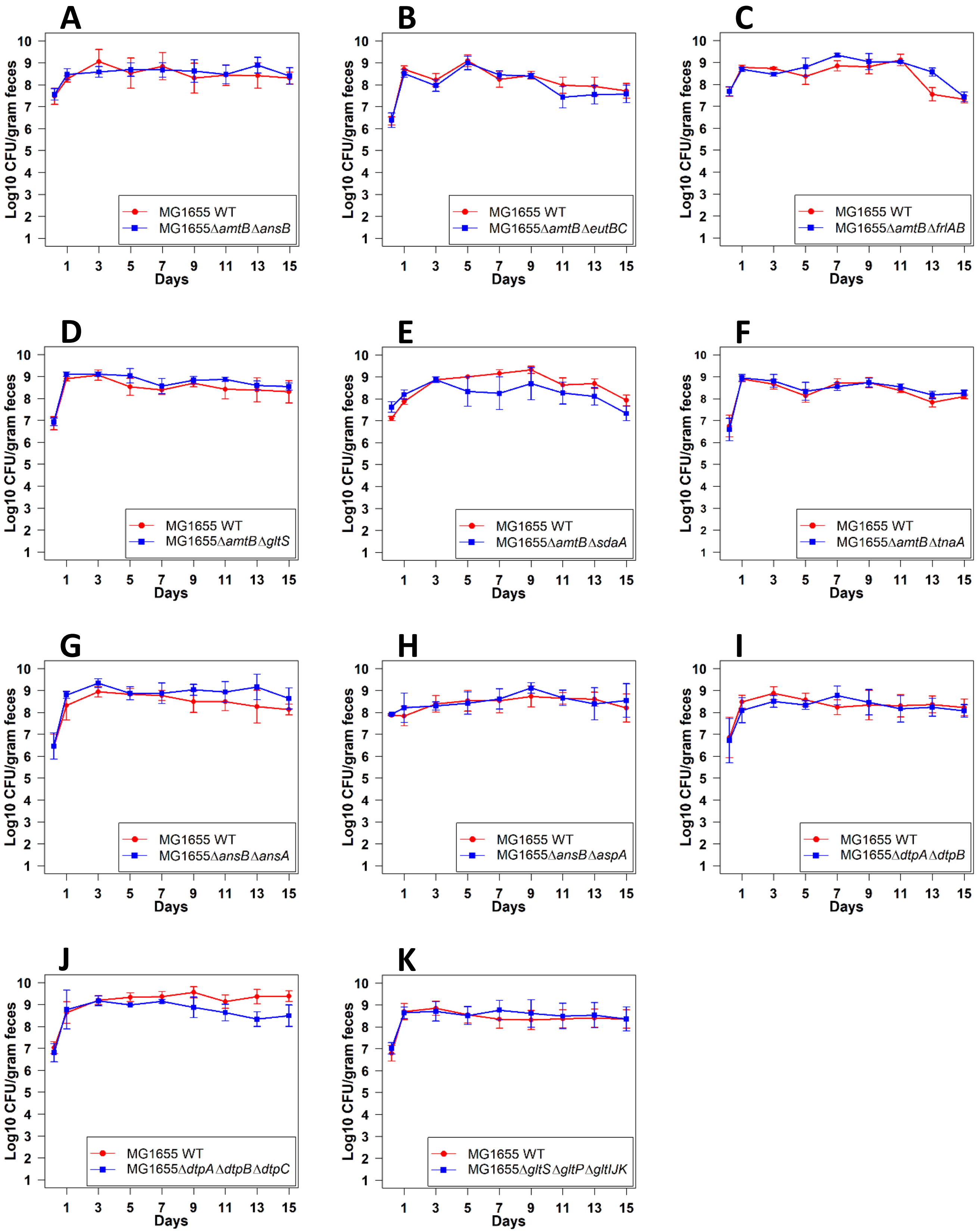
A
Log10 CFU/gram feces
Days
MG1655 WT
MG1655ΔamtBΔansB
B
Log10 CFU/gram feces
Days
MG1655 WT
MG1655ΔamtBΔeutBC
C
Log10 CFU/gram feces
Days
MG1655 WT
MG1655ΔamtBΔfrlAB
D
Log10 CFU/gram feces
Days
MG1655 WT
MG1655ΔamtBΔgltS
E
Log10 CFU/gram feces
Days
MG1655 WT
MG1655ΔamtBΔsdaA
F
Log10 CFU/gram feces
Days
MG1655 WT
MG1655ΔamtBΔtnaA
G
Log10 CFU/gram feces
Days
MG1655 WT
MG1655ΔansBΔansA
H
Log10 CFU/gram feces
Days
MG1655 WT
MG1655ΔansBΔaspA
I
Log10 CFU/gram feces
Days
MG1655 WT
MG1655ΔdtpAΔdtpB
J
Log10 CFU/gram feces
Days
MG1655 WT
MG1655ΔdtpAΔdtpBΔdtpC
K
Log10 CFU/gram feces
Days
MG1655 WT
MG1655ΔgltSΔgltPΔgltIJK

Figure S5. Competitive colonization of the mouse intestine by *E. coli* MG1655 Str^R Nal^R Wild Type and a double, triple or quadruple deletion mutant. One or two sets of three CD-1 male mice were fed 10^5 CFU of *E. coli* MG1655 Str^R Nal^R Wild Type and 10^5 CFU of *E. coli* mutant. At indicated times, samples were collected, homogenized, diluted, and plated. Error bars represent standard errors of the pooled data of $\log_{10}$ mean CFU per gram of feces. **A.** competition between *E. coli* MG1655 Str^R Nal^R Wild Type and *E. coli* MG1655 Str^R Δ*amtB*Δ*ansB*::*cam* **B.** competition between *E. coli* MG1655 Str^R Nal^R Wild Type and *E. coli* MG1655 Str^R Δ*amtB*Δ*eutBC*::*cam* **C.** competition between *E. coli* MG1655 Str^R Nal^R Wild Type and *E. coli* MG1655 Str^R Δ*amtB*Δ*frlAB*::*cam* **D.** competition between *E. coli* MG1655 Str^R Nal^R Wild Type and *E. coli* MG1655 Str^R Δ*amtB*Δ*gltS*::*cam* **E.** competition between *E. coli* MG1655 Str^R Nal^R Wild Type and *E. coli* MG1655 Str^R Δ*amtB*Δ*sdaA*::*cam* **F.** competition between *E. coli* MG1655 Str^R Nal^R Wild Type and *E. coli* MG1655 Str^R Δ*amtB*Δ*tnaA*::*cam* **G.** competition between *E. coli* MG1655 Str^R Nal^R Wild Type and *E. coli* MG1655 Str^R Δ*ansB*Δ*ansA*::*cam.* **H.** competition between *E. coli* MG1655 Str^R Nal^R Wild Type and *E. coli* MG1655 Str^R Δ*ansB*Δ*aspA*::*cam* **I.** competition between *E. coli* MG1655 Str^R Nal^R Wild Type and *E. coli* MG1655 Str^R Δ*dtpA*Δ*dtpB*::*cam* **J.** competition between *E. coli* MG1655 Str^R Nal^R Wild Type and *E. coli* MG1655 Str^R Δ*dtpA*Δ*dtpB*Δ*dtpC*::*cam* **K.** competition between *E. coli* MG1655 Str^R Nal^R Wild Type and *E. coli* MG1655 Str^R Δ*gltS*Δ*gltS*Δ*gltIJK*::*cam*

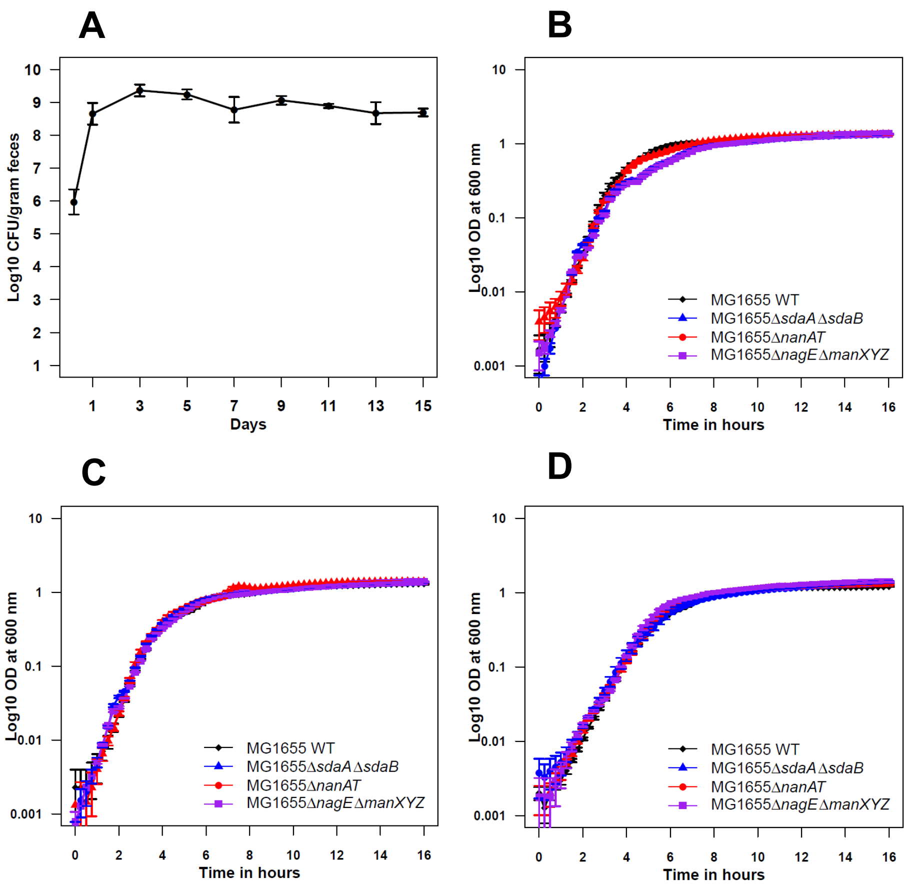

Figure S6. In vitro and in vivo growth of *E. coli* MG1655 Str^R Δ*sdaA* Δ*sdaB::cam* A. Monocolonization of *E. coli* MG1655 Str^R Δ*sdaA* Δ*sdaB::cam* in the mouse intestine. **B, C and D.** Growth of *E. coli* MG1655 Str^R Nal^R wild type, *E. coli* MG1655 Str^R Δ*sdaA* Δ*sdaB::cam*, *E. coli* MG1655 Str^R Δ*nanAT::kan and E. coli* MG1655 Str^R Δ*nagE* Δ*manXYZ::kan* in LB medium (**B**), LB medium containing 0.2% glucose (**C**) and LB medium containing 0.2% phosphoenol pyruvate (**D**).

References

1. Sander T, Farke N, Diehl C, Kuntz M, Glatter T, Link H. 2019. Allosteric Feedback Inhibition Enables Robust Amino Acid Biosynthesis in *E. coli* by Enforcing Enzyme Overabundance. Cell Syst 8:66-75 e8.

2. Keseler IM, Gama-Castro S, Mackie A, Billington R, Bonavides-Martinez C, Caspi R, Kothari A, Krummenacker M, Midford PE, Muniz-Rascado L, Ong WK, Paley S, Santos-Zavaleta A, Subhraveti P, Tierrafria VH, Wolfe AJ, Collado-Vides J, Paulsen IT, Karp PD. 2021. The EcoCyc Database in 2021. Front Microbiol 12:711077.
